# Effects of diagnostic regions on facial emotion recognition: The moving window technique

**DOI:** 10.3389/fpsyg.2022.966623

**Published:** 2022-09-08

**Authors:** Minhee Kim, Youngwug Cho, So-Yeon Kim

**Affiliations:** ^1^Department of Psychology, Duksung Women’s University, Seoul, South Korea; ^2^Department of Computer Science, Hanyang University, Seoul, South Korea

**Keywords:** emotion recognition, facial expression, face processing strategy, diagnostic region, moving window technique

## Abstract

With regard to facial emotion recognition, previous studies found that specific facial regions were attended more in order to identify certain emotions. We investigated whether a preferential search for emotion-specific diagnostic regions could contribute toward the accurate recognition of facial emotions. Twenty-three neurotypical adults performed an emotion recognition task using six basic emotions: anger, disgust, fear, happiness, sadness, and surprise. The participants’ exploration patterns for the faces were measured using the Moving Window Technique (MWT). This technique presented a small window on a blurred face, and the participants explored the face stimuli through a mouse-controlled window in order to recognize the emotions on the face. Our results revealed that when the participants explored the diagnostic regions for each emotion more frequently, the correct recognition of the emotions occurred at a faster rate. To the best of our knowledge, this current study is the first to present evidence that an exploration of emotion-specific diagnostic regions can predict the reaction time of accurate emotion recognition among neurotypical adults. Such findings can be further applied in the evaluation and/or training (regarding emotion recognition functions) of both typically and atypically developing children with emotion recognition difficulties.

## Introduction

The ability to accurately recognize emotions through facial expressions is an important social competency for participation in social interactions. In everyday life, facial expressions of emotion are one of the most important means of emotional communication, as referencing others’ facial expressions can be a cue for choosing appropriate social behaviors. Both intentionally and automatically, people convey their emotions through facial expressions, perceive others’ emotional states and interpret behavioral intentions through facial expressions (Horstmann, 2003). Thus, it is important to recognize emotions in facial expressions in order to ensure successful social functioning.

Facial expressions of emotion are expressed through various facial motions. According to Ekman et al. (1972) theory of basic emotion, six basic emotions (happiness, anger, disgust, fear, sadness, and surprise) are universally found across cultures (Ekman et al., 1987). These emotions can be expressed and recognized through an emotion-specific configuration of facial muscle movements (Ekman and Friesen, 1978; Cohn et al., 2007; Wolf, 2015). Accordingly, the accurate recognition of particular emotions in facial expressions can be associated with the accurate recognition of emotion-specific facial movement patterns.

Previous studies have suggested that the eye and mouth regions are the most expressive regions of facial features. In a study examining sex differences in emotion recognition, women showed more accurate and faster recognition of facial expressions than men, and women’s gaze toward the eye region was correlated with their accuracy and faster responses (Hall et al., 2010). In contrast, a study where participants viewed random facial parts while other visual information of facial stimuli was restricted suggested that the mouth region produced the most informative facial cues for recognizing emotions in both dynamic and static facial expressions (Blais et al., 2012). Furthermore, a study on individuals with autism spectrum disorder, who commonly revealed social communication deficits, showed a reduced fixation time for the eye region compared to the typical development group, and their increased eye-gaze pattern toward the mouth region was associated with their social competence (Klin et al., 2002; Norbury et al., 2009).

However, there have been some disagreement regarding which facial parts are relatively more important for emotion recognition: the eye or mouth region. Therefore, an alternative explanation is proposed, which claims that the relative importance of the facial regions for accurate emotion recognition differs depending on the type of emotion. That is, the importance of particular facial features (i.e., either of the eye or the mouth region) for emotion recognition functions may not be common across emotions but may differ depending on the type of emotion (Eisenbarth and Alpers, 2011; Schurgin et al., 2014; Wegrzyn et al., 2017). Such emotion-specific facial region for emotion recognition is called the diagnostic region of emotion, in which emotion-specific information is contained the most. Several studies have supported this idea and shown that preferential processing of the diagnostic region is important for emotion recognition (Birmingham et al., 2013; Neta and Dodd, 2018; Bodenschatz et al., 2019).

Prior studies on diagnostic regions have identified consistent results regarding basic emotions. For anger, fear, and sadness, the eye region is considered to express more emotional information than the other facial parts, while the mouth region is considered to express more emotional information related to happiness and disgust. Regarding the emotion of surprise, inconsistent results across studies have shown that the diagnostic regions for surprise could be the eye, mouth, or both regions (Kestenbaum, 1992; Eisenbarth and Alpers, 2011; Tanaka et al., 2012; Birmingham et al., 2013; Schurgin et al., 2014; Wegrzyn et al., 2017; Neta and Dodd, 2018; Bodenschatz et al., 2019).

For example, Schurgin et al. (2014) employed an eye-tracking method to examine the eye-gaze pattern for each emotion during an emotion recognition task. The results showed that, for fear, anger, and sadness, the fixation time for the eye region was significantly greater than the mean fixation time, while the fixation time for the mouth region was shorter. In contrast, for happiness and disgust, the fixation time for the mouth region was significantly greater than the mean fixation time, while the fixation time for the eye region was shorter. Another study conducted by Wegrzyn et al. (2017) illustrated similar results by applying a method in which the facial expressions of emotion were mapped, and participants were presented with a randomly opening sequence of tiles that covered facial expressions. To assess which facial parts were the most important for decoding emotions, the researchers calculated each tile’s weight for each facial expression based on the number of tiles opened until the viewers recognized the emotion. The results demonstrated that the eye regions provided the most diagnostic value for emotion identification for fear, anger, and sadness. However, for happiness and disgust, the mouth region provided the most diagnostic value. Furthermore, the researchers found that both the eye and mouth regions significantly contributed to helping participants recognize surprise. Another study using the Moving Window Technique (MWT) also reported similar results regarding the diagnostic regions for facial emotions, especially for anger, fear, happiness, and disgust. The MWT presents viewers with a blurred face stimulus and provides them with a mouse-controlled window through which they can explore facial parts. Researchers found that participants (in the age group of 5 years to young adulthood) revealed emotion-specific diagnostic regions for anger, fear, happiness, and disgust across various age groups (Birmingham et al., 2013).

The diagnostic region is defined by facial features that contain the most emotional information of the facial features. Given that most studies determined emotion-specific diagnostic regions by examining which facial features were gazed at or utilized to decode a particular emotion, the exploration of emotion-specific diagnostic regions may be related to accurate emotion recognition. Accordingly, previous studies have attempted to clarify the relationship between these diagnostic regions and the accuracy of emotion recognition. However, this relationship has not been well determined. A few past studies have shown that the preferential exploration of the diagnostic regions is related to accurate emotion recognition. For instance, regarding the recognition of happiness, disgust, and anger, Schurgin et al. (2014) found that facial emotions were more accurately identified when the relevant emotion-specific diagnostic regions were visible compared to when these regions were covered. However, this study could not show the contribution of active exploration of the diagnostic regions toward emotion recognition accuracies, because they presented diagnostic regions passively to the participants. Likewise, Birmingham et al. (2013) found that the more exploration of the eye region relative to the mouth region, the higher accuracy rates for anger and fear emotions. In contrast, the authors also reported that the more exploration of the mouth region relative to the eye region, the higher accuracy rates for the disgust emotion. However, Birmingham et al. (2013) found only partial evidence on the relationships between the search patterns on diagnostic regions and emotion recognition performance. That is, the researchers showed only weak correlations between the exploration patterns on the diagnostic regions and emotion recognition accuracies for some emotions (i.e., anger, fear, and disgust). Also, they did not report separate results for each age group (i.e., children and adults) despite the fact that there were age differences in emotion recognition accuracies and searching patterns on the diagnostic regions. Furthermore, other studies have failed to derive such evidence to support the statement that a preferential search for diagnostic regions contributes to emotion recognition. In fact, others have found contradictory associations with previously identified emotion-specific diagnostic regions. For example, a longer fixation of the gaze on the mouth region was significantly correlated with recognition of anger, and a longer gaze on the eye region was coupled with recognition of disgust (Green and Guo, 2018; Duran and Atkinson, 2020; Yitzhak et al., 2020).

As such, whether the exploration of diagnostic regions enhances the facial emotion recognition process has not been consistently proven, although the existence of such emotion recognition diagnostic regions has been well supported with research using various experimental methods. One of the possible reasons for such contradictory findings is the methodological differences across studies. The eye-tracking method has been widely applied because it can detect eye-gaze patterns toward each facial region. However, typical parameters used in the eye-tracking method can only reflect information from foveal vision, whereas extrafoveal information is uncontrolled. Given that extrafoveal visual information can also guide attention (Cimminella et al., 2020) and influence the classification of emotions (Atkinson and Smithson, 2020), eye-tracking may not be a sufficient method for identifying which facial region is being selectively explored when specific emotions are being recognized. Other studies have attempted to avoid this problem by restricting the available visual information. For example, the Bubbles technique utilizes sparsely spread bubbles through which participants view the partially revealed facial regions (Gosselin and Schyns, 2001; Zhang et al., 2010; Blais et al., 2017), and a method of mapping the facial expressions sequentially reveals the tiles covering the face (Wegrzyn et al., 2017). However, such methods could complicate the process of capturing the contribution of diagnostic regions to emotion recognition, as they do not allow viewers’ active exploration of facial areas but restrict visual information passively. Therefore, the present study adopted the MWT to examine participants’ attention fixation-based search patterns on faces. The MWT presents blurred faces and provides a window for exploring the facial regions. The window, which is controlled by viewers, enables the measurement of overt orienting, and the blurring procedure controls the influences of unmeasured information from extrafoveal vision.

Another possible reason for the mixed findings regarding the effects of diagnostic regions on emotion recognition may be related to ceiling effects or threshold issues. That is, neurotypical young adults without any notable difficulties regarding facial emotion recognition might not reveal significant differences in emotion recognition accuracy, as these individuals’ general performance on facial emotion recognition can be too high (e.g., ceiling effects) when there is a sufficient intensity in the emotions. Instead, a rapid—or even automatic—search for diagnostic regions may play a role in determining the efficiency of accurate facial emotion recognition. To process complex visual stimuli, people apply visual attention in order to selectively focus on what is important for recognizing the stimuli. Selective attention helps us properly choose important visual features and enables efficient perceptual processing. When perceiving emotion on a face, the course of the attentional process (i.e., selectively focusing on a key diagnostic region) can contribute to the efficiency of emotion recognition. Thus, the present study aimed to examine whether exploration of emotion-specific diagnostic regions predicts the efficiency of correct responses to specific facial emotions.

To expand the findings of previous research that examined the effects of diagnostic regions for only certain emotions (Birmingham et al., 2013, 2018; Schurgin et al., 2014), the current study included all six basic emotions (i.e., anger, disgust, fear, happiness, sadness, and surprise). Based on previously identified diagnostic regions (Birmingham et al., 2013; Schurgin et al., 2014; Wegrzyn et al., 2017; Neta and Dodd, 2018; Bodenschatz et al., 2019), the aim of the current study was to discover whether the emotion-specific diagnostic regions would predict the efficiency of the correct recognition of facial emotion for the six basic emotions. Specifically, we hypothesized that searching for the emotion-specific diagnostic regions would significantly explain the efficiency of the facial emotion recognition. That is, for anger, fear, and sadness, we hypothesized that the exploration patterns for the eye regions would predict a fast reaction time (RT) for accurate emotion recognition. For happiness and disgust, the exploration patterns for the mouth region would predict a fast RT for accurate emotion recognition. Furthermore, exploration of both the eye and mouth regions would predict a fast RT for the emotion of surprise. To test these hypotheses, this study employed the MWT to allow for active exploration of the facial features while controlling for unattended visual information. As the participants moved the window over the face stimuli, their movement patterns were recorded. Based on previous findings on emotion recognition, the eye and mouth regions were defined as the regions of interest (ROIs). For each ROI, (1) the extent to which the region was explored (i.e., spent time) and (2) where the last exploration occurred (i.e., the last ROI) were recorded and assessed.

## Materials and methods

### Participants

Twenty-three undergraduate students aged 18–24 years (mean = 20.6, SD = 1.7) were recruited (all female) from Duksung Women’s University. All the participants were Korean and reported normal or corrected-to-normal vision. The participants were compensated with 10,000 won for their participation, and informed consent was obtained prior to the experiment. The study was conducted in accordance with the ethics committee approval of Duksung Women’s University.

### Stimuli

**Figure 1:**
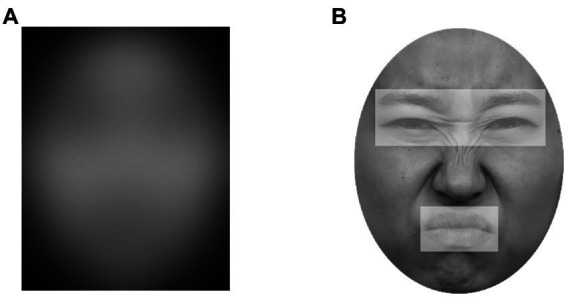
Examples of facial stimuli and ROI in the experiment. **(A)** Blurred image in the MWT. **(B)** An example of the eye and mouth regions. Adapted from the Korean Facial Expressions of Emotion (KOFEE) (Park et al., 2011), with permission from Dr. Suk Kyoon An.

Eight faces (four females and four males) depicting each basic emotion (happiness, anger, disgust, fear, sadness, and surprise) were selected from the Korean Facial Expressions of Emotion (KOFEE. Park et al., 2011). A total of 48 images were blurred with a Gaussian filter (radius = 90 pixels), modified in grayscale, and modified to fit a round shape on a black background in Adobe Photoshop CS5 (Adobe Systems Inc., San Jose, CA, United States; Figure 1A). Filtering was adjusted so that the emotion could not be identified without a window based on a previous study (Birmingham et al., 2013). Stimuli were displayed at 750 × 950 pixels in size corresponding to 18 × 23 degrees of visual angle at a viewing distance of 60 cm. Based on the results of a pilot study, stimuli that were recognized accurately over 70% of the time were included for each emotion, except for fear (see “Pilot Study” for details).

### Procedure

The participants were seated in front of a computer screen with a handheld mouse and keyboard in a separate room. Before the experimental trials commenced, six faces corresponding to each emotion were presented individually to the participants to help them familiarize themselves with the stimuli and ensure that they understood the labels of each emotion. Participants were then given verbal instructions, and six practice trials were performed. The facial expressions used in the familiarization and practice trials were different from those used in the experimental trials. Each experimental block consisted of 48 trials that were commenced in a random order, and overall, two blocks were conducted.

**Figure 2:**
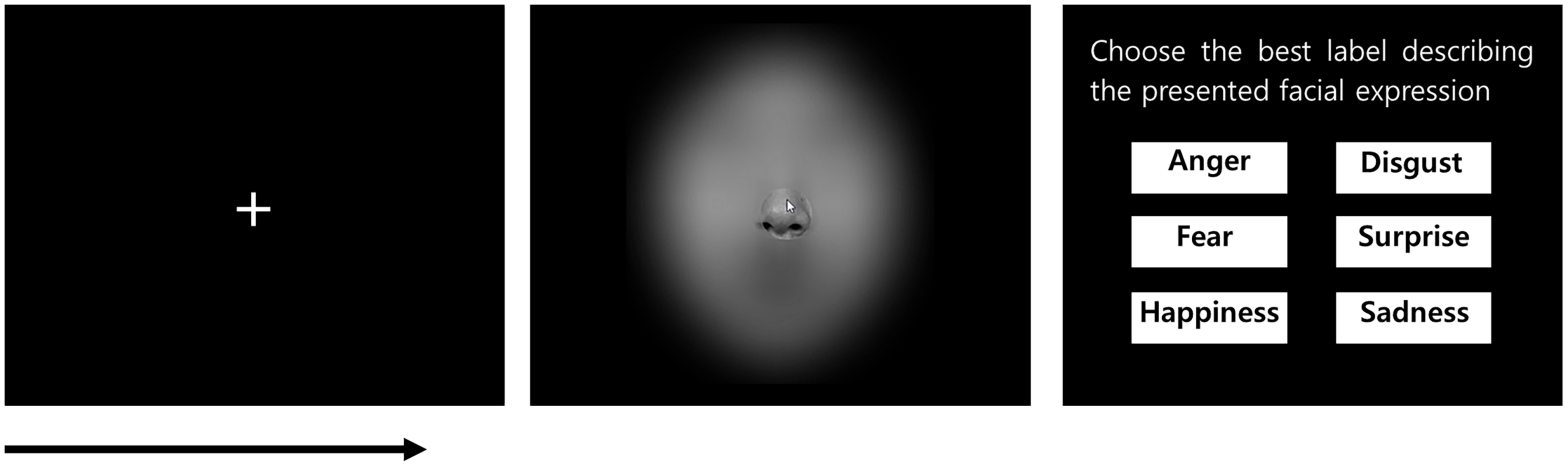
Example display of an experimental trial in the emotion recognition task. Adapted from the Korean Facial Expressions of Emotion (KOFEE) (Park et al., 2011), with permission from Dr. Suk Kyoon An.

Each experimental trial started as follows: participants were presented with a central white fixation on a black background until they pressed the spacebar when they were ready. Subsequently, a blurred image of a face was presented with a window size of 160 × 160 pixels. The window size ensured that only one facial feature was revealed at a time. The window was fully controlled by a handheld mouse so that the participants could move the window to recognize the emotion. Participants were instructed to decode the emotion by freely moving the window as fast and accurately as possible and to press the spacebar to respond as soon as they recognized the emotion. As the participants pressed the spacebar, six emotion labels were displayed, and participants were instructed to choose the best label that explained the facial expression by clicking a mouse. If a response was made, the next trial started with a white fixation (Figure 2). For each trial, coordinate value of the window, response time (RT), which represented the time (second) in between the presentation of the face and pressing the spacebar, and accuracy of emotion response were collected.

### Parameters and analysis

The mouse was coordinated with the center of the window, and the real-time coordinate value of the mouse was acquired at a rate of 60 Hz. Two parameters were computed based on the participants’ moving pattern for the mouse over the two preset ROIs (i.e., eye and mouth regions) for each facial stimulus (Figure 1B). Based on a previous study (Birmingham et al., 2013), *Spent Time* was calculated using the following steps: (1) Multiplying the overlapping pixels between the window and ROI with the number of time samples (each time sample lasted approximately 17 ms). (2) Normalizing this value by the size of the pixels for each ROI to correct the difference in sizes between the two ROIs. (3) The proportion of the value for each ROI across the whole face is calculated. Spent time reflects how long and how much each facial region is explored for a specific emotion. Furthermore, *Last ROI* was calculated based on the ROI explored for approximately 50 ms (for three time samples) before finishing the exploration. The exploration frequency was computed for each ROI for the last ROI based on the position of the mouse at each time sample. Because the participants ended the trials by themselves, the last ROI reflected the region where the specific emotion recognition was made.

We aimed to explain the efficiency of correct emotion recognition using search patterns for emotion diagnostic regions; the abovementioned two parameters (i.e., spent time and last ROI) were computed for each ROI in the correct trials and averaged across participants for each emotion. To find the explanatory variable for the regression model, we conducted a 6 (emotion: anger, disgust, fear, happiness, sadness, surprise) × 2 (ROI: eye region, mouth region) repeated measures analysis of variance (ANOVA) on spent time and last ROI separately. We then set two regression models: one for variables that showed significant effects in ANOVAs and another for generally considered diagnostic regions based on previous findings (Eisenbarth and Alpers, 2011; Birmingham et al., 2013, 2018; Schurgin et al., 2014; Wegrzyn et al., 2017; Bodenschatz et al., 2019). We conducted regression analyses for each model to find variables explaining RTs and determined the common significant explanatory variables in both models as significant factors explaining fast RT.

### Pilot study

A pilot study was conducted to validate these stimuli. Twelve participants (mean age = 25 years, female = 9), who did not participate in the actual MWT experiment, performed an emotion categorization task. Seventy-eight images of facial expressions (anger = 13, disgust = 11, fear = 11, happiness = 15, sadness = 13, surprise = 15, size 503 × 637 pixels) from the Korean Facial Expressions of Emotion (KOFEE. Park et al., 2011) were randomly displayed in grayscale at the center of the screen. The participants were instructed to indicate which emotion the presented face was depicting. Based on the accuracy data for recognizing each emotion (see Table 1 for the mean accuracy and reaction time), faces with higher than 70% of accuracy rate were included in Study 1, except for fear. Since the accuracy for recognition of fear has been reported to be relatively lower than other facial emotions in Koreans (Kim et al., 2017c; Chung et al., 2019), faces with a recognition accuracy over 50% were included in the fear emotion.

**Table 1:** Mean (standard deviation) accuracy and reaction time for each emotion in the “Pilot study.”

	**Anger**	**Disgust**	**Fear**	**Happiness**	**Sadness**	**Surprise**
**Accuracy (%)**	84.62 (17.36)	78.03 (21.74)	48.48 (30.85)	88.33 (23.29)	92.31 (8.68)	95.56 (5.19)
**Reaction Time (s)**	3.50 (1.46)	4.44 (2.40)	4.43 (2.96)	2.75 (1.16)	2.81 (0.67)	2.39 (0.80)

## Results

### Accuracy and reaction time

Accuracies and reaction times (RTs) for emotion recognition were averaged across participants for each emotion. The mean accuracies and RT data are listed in Table 2. A one-way ANOVA on accuracy revealed a significant main effect of emotion [*F*(2.76, 60.74) = 23.43, *p* < 0.001, $\eta_{p}{}^{2}$ = 0.52]. *Post-hoc* paired *t*-tests with Bonferroni correction for accuracy revealed that happiness and surprise were recognized more accurately than anger [*t*(22) = −3.64, *p* = 0.001; *t*(22) = −4.7, *p* < 0.001], disgust [*t*(22) = −5.41, *p* < 0.001; *t*(22) = −5.21, *p* < 0.001] and fear [*t*(22) = −7.5, *p* < 0.001; *t*(22) = −7.92, *p* < 0.001]. Furthermore, sadness was recognized more accurately than disgust [*t*(22) = −3.81, *p* < 0.001] and fear [*t*(22) = −6.25, *p* < 0.001].

**Table 2:** Mean (standard deviation) accuracy and reaction time for each emotion in the emotion recognition task.

	**Anger**	**Disgust**	**Fear**	**Happiness**	**Sadness**	**Surprise**
**Accuracy (%)**	91.58 (6.42)	87.23 (9.70)	78.26 (12.90)	97.55 (5.88)	96.20 (5.88)	98.10 (3.50)
**Reaction Time(s)**	4.01 (1.76)	3.65 (1.33)	4.74 (1.99)	3.00 (0.89)	3.70 (1.16)	3.20 (1.14)

The same analyses conducted on the RT data for trials with correctly recognized emotions revealed a significant main effect of emotion [*F*(2.93, 64.51) = 16.87, *p* < 0.001, $\eta_{p}{}^{2}$ = 0.43]. *Post-hoc* *t*-tests showed that happiness was identified faster than other emotions [disgust: *t*(22) = 4.17, *p* < 0.001; sadness: *t*(22) = −5.3, *p* < 0.001; anger: *t*(22) = 4.2, *p* < 0.001; fear: *t*(22) = 5.66, *p* < 0.001] except for surprise. Surprise was recognized faster than sadness [*t*(22) = 3.36, *p* = 0.003], anger [*t*(22) = 3.69, *p* = 0.001], and fear [*t*(22) = 5.44, *p* < 0.001]. Finally, fear was recognized more slowly than the other emotions [sadness: *t*(22) = 3.89, *p* = 0.001; disgust: *t*(22) = −4.47, *p* < 0.001], except for anger.

Compared to the results of the pilot study, recognition accuracies were slightly higher, and RTs were slightly slower for all emotions in the MWT. These results imply a trade-off between accuracy and RT in the MWT compared with the traditional emotion recognition task, given that the MWT provides participants with more detailed explorations of facial expressions. The overall recognition patterns for each emotion were similar in both tasks.

### Spent time and last ROI

The 2 (ROI) × 6 (Emotion) repeated measures ANOVAs on spent time and last ROI revealed significant main effects of emotion [*F*(5, 110) = 14.28, *p* < 0.001, $\eta_{p}{}^{2}$ = 0.39; *F*(5, 110) = 13.87, *p* < 0.001, $\eta_{p}{}^{2}$ = 0.39] and ROI [*F*(1, 22) = 27.4, *p* < 0.001, $\eta_{p}{}^{2}$ = 0.56; *F*(1, 22) = 30.06, *p* < 0.001, $\eta_{p}{}^{2}$ = 0.58]. Furthermore, the interaction between emotion and ROI was significant [*F*(5, 110) = 8.65, *p* < 0.001, $\eta_{p}{}^{2}$ = 0.28; *F*(5, 110) = 12.55, *p* < 0.001, $\eta_{p}{}^{2}$ = 0.36].

**Figure 3:**
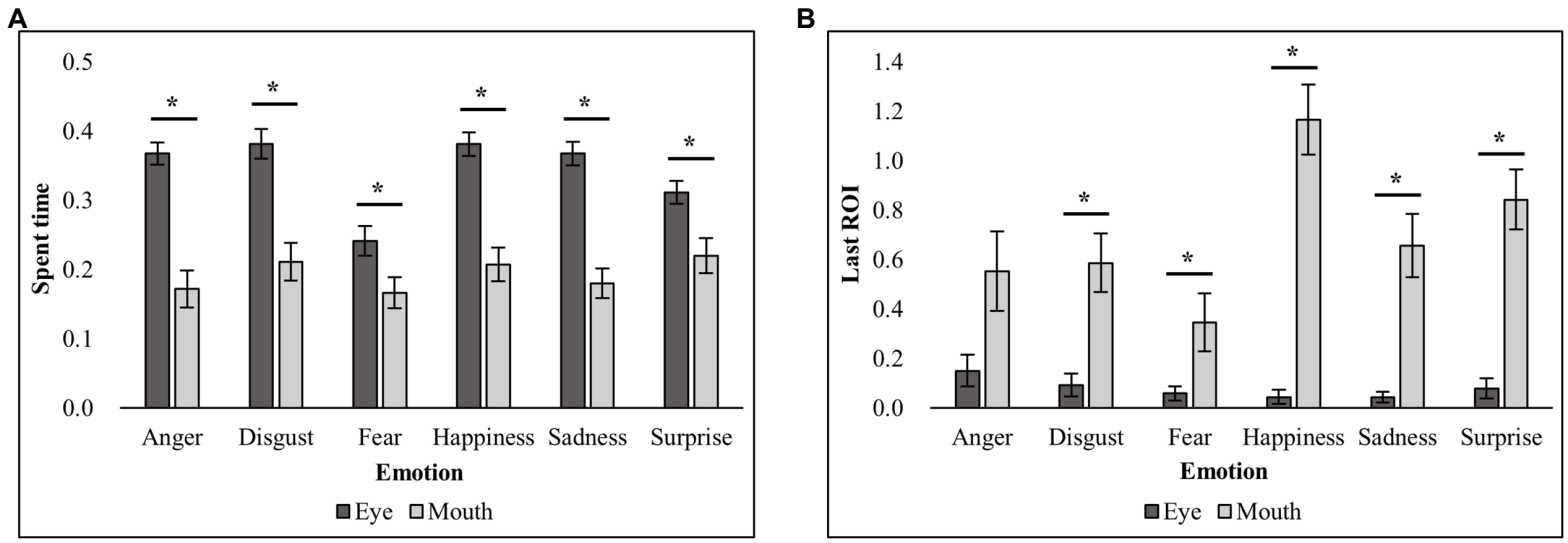
Mean spent time and last ROI for each emotion and ROI. **(A)** The spent time (%) for each emotion and ROI. **(B)** The last ROI (frequency) for each emotion and ROI. ^*^ indicates the significant results in *post-hoc* paired *t*-tests.

To find the explanatory variables for the following regression analyses, *post-hoc* *t*-tests with Bonferroni correction on the interaction effect were conducted. For each dependent variable (spent time and last ROI), comparisons between the eye and mouth regions were conducted for each emotion. *Post-hoc* *t*-tests for the spent time showed that the spent time for the eye region was significantly greater than the spent time for the mouth region for all emotions [anger: *t*(22) = 6, *p* < 0.001; disgust: *t*(22) = 4.4, *p* < 0.001; fear: *t*(22) = 2.79, *p* < 0.05; happiness: *t*(22) = 4.89, *p* < 0.001; sadness: *t*(22) = 6.32, *p* < 0.001; surprise: *t*(22) = 2.91, *p* < 0.05]. *Post-hoc* *t*-tests for the last ROI showed that the last ROI for the mouth region was significantly greater than the last ROI for the eye region for all emotions except for anger [disgust: *t*(22) = 4.15, *p* < 0.001; fear: *t*(22) = 2.63, *p* < 0.05; happiness: *t*(22) = 7.89, *p* < 0.001; sadness: *t*(22) = 4.75, *p* < 0.001; surprise: *t*(22) = 6.43, *p* < 0.001]. Figure 3 illustrates the results of the analyses on each dependent variable (spent time and last ROI) for each ROI and emotion.

### Regression analysis

We conducted a series of linear regression analyses to test our hypothesis that the exploration of diagnostic regions would predict efficient emotion recognition (i.e., faster RTs for correct trials). Two regression models were constructed based on (1) the results of the current study and (2) previously identified emotion-specific diagnostic regions based on previous studies (i.e., variables related to the eye region for anger, fear, and sadness; variables related to the mouth region for disgust and happiness; variables related to both the eye and mouth regions for surprise; Eisenbarth and Alpers, 2011; Birmingham et al., 2013, 2018; Schurgin et al., 2014; Wegrzyn et al., 2017; Bodenschatz et al., 2019; Table 3).

**Table 3:** Regression models conducted in the current study.

**Dependent variable**	**Regressors based on current study**	**Regressors based on prior findings**
**Anger RT**	Spent time for eye	Spent time for eye, last ROI for eye
**Disgust RT**	Spent time for eye, last ROI for mouth	Spent time for mouth, last ROI for mouth
**Fear RT**	Spent time for eye, last ROI for mouth	Spent time for eye, last ROI for eye
**Happiness RT**	Spent time for eye, last ROI for mouth	Spent time for mouth, last ROI for mouth
**Sadness RT**	Spent time for eye, last ROI for mouth	Spent time for eye, last ROI for eye
**Surprise RT**	Spent time for eye, last ROI for mouth	Spent time for eye, spent time for mouth, last ROI for eye, last ROI for mouth

#### Regression analysis: Based on the current findings

Variables which showed significant differences in the *post-hoc* *t*-tests were included as explanatory factors in the regression models based on the findings from current study (Table 3, the left column). For anger, a regression analysis was conducted to predict RTs with the spent time for the eye region as a regressor. The results showed that the spent time for the eye regions significantly explained the variances in the RTs for anger recognition [*F*(2, 20) = 5.77, $R^{2}$ *= 0.*22, *β* = −10.4, *t*(22) = −2.4, *p* < 0.05]. For the other emotions, multiple regression analyses were conducted with the spent time for the eye region and the last ROI for the mouth region as regressors and RTs for each emotion as dependent variables. For disgust, the last ROI for the mouth region was revealed to be a significant factor in explaining variances in RTs [*F*(2, 20) = 11.08, $R^{2}$ = 0.53, *β* = −1.62*, t*(22) = −4.45, *p* < 0.001]. For fear, both the spent time for the eye region and the last ROI for the mouth region significantly explained the variances in RTs [*F*(2, 20) = 11.72, $R^{2}$ = 0.54, *β* = −8.74, *t*(22) = −2.74, *p* = 0.01; *β* = −1.46, *t*(22) = −2.49, *p* < 0.05]. For happiness, the last ROI for the mouth region was a significant factor for explaining RTs in recognizing emotions [*F*(2, 20) = 15.12, $R^{2}$ = 0.60, *β* = −1.02, *t*(22) = −5.27, *p* < 0.001]. For sadness, the last ROI for the mouth region significantly predicted faster RTs for sadness recognition [*F*(2, 20) = 8.95, $R^{2}$ = 0.47, *β* = −1.37, *t*(22) = −4.18*, p* < 0.001]. For the surprise emotion, the results showed that both the spent time for the eye region and the last ROI for the mouth region significantly explained variances in RTs [*F*(2, 20) = 20.4, $R^{2}$ = 0.67, *β* = −4.41, *t*(22) = −2.33, *p* < 0.05; *β* = −1.53, *t*(22) = −6.06, *p* < 0.001].

#### Regression analysis: Based on findings from prior studies

With explanatory variables based on the previous studies on the diagnostic regions, we conducted multiple regression analyses for each emotion (Table 3, the right column). For anger, a regression analysis with both the spent time and last ROI for the eye region as regressors conducted to predict RTs. The spent time for the eye regions in the regression model showed significant effects for explaining RTs in recognizing anger [*F*(2, 20) = 3.65, $R^{2}$ = 0.27, *β* = −2.4, *t*(22) = −2.4, *p <* 0.05]. For disgust, a regression analysis was conducted to predict the RTs with the spent time and last ROI for the mouth region. The results revealed that both the spent time for the mouth region and the last ROI for the mouth were significant factors in explaining RTs [*F*(2, 20) = 14.99, $R^{2}$ = 0.6, *β* = −4.19, *t*(22) = −2.23, *p* < 0.05; *β* = −1.04, *t*(22) = −2.42, *p* < 0.05]. For fear, a multiple regression analysis was conducted with the spent time and last ROI for the eye region as regressors on RTs. The results showed that the spent time for the eye region was significant [*F*(2, 20) = 9.31, $R^{2}$ = 0.48, *β* = −10.84, *t*(22) = −3.44, *p <* 0.05]. For happiness, a regression analysis was conducted with the spent time and last ROI for the mouth region as regressors to predict the RT patterns. In the results, the last ROI for the mouth region was a significant factor for explaining RTs in recognizing happy emotions [*F*(2, 20) = 15.1, $R^{2}$ = 0.60, *β* = −0.96, *t*(22) = −2.86, *p* = 0.01]. For sadness, a regression analysis with the spent time and last ROI for eye region as regressors was conducted on RTs, and no significant effect was found. Although the surprise emotion has shown inconsistent results with regard to diagnostic regions in prior studies, Wegrzyn et al. (2017) suggested that both the eye and mouth regions were diagnostic for recognition of the surprise emotion. Thus, for the surprise emotion, a multiple regression analysis with all four variables (i.e., the spent time and the last ROI for each ROI) as regressors was conducted to predict RTs. The results revealed that both the spent time for the eye region and the last ROI for the mouth region significantly explained faster RTs for recognizing the surprise emotion [*F*(2, 20) = 20.4, $R^{2}$ = 0.67, *β* = −4.41, *t*(22) = −2.33, *p* < 0.05; *β* = −1.53, *t* (22) = −6.06, *p* < 0.001].

**Figure 4:**
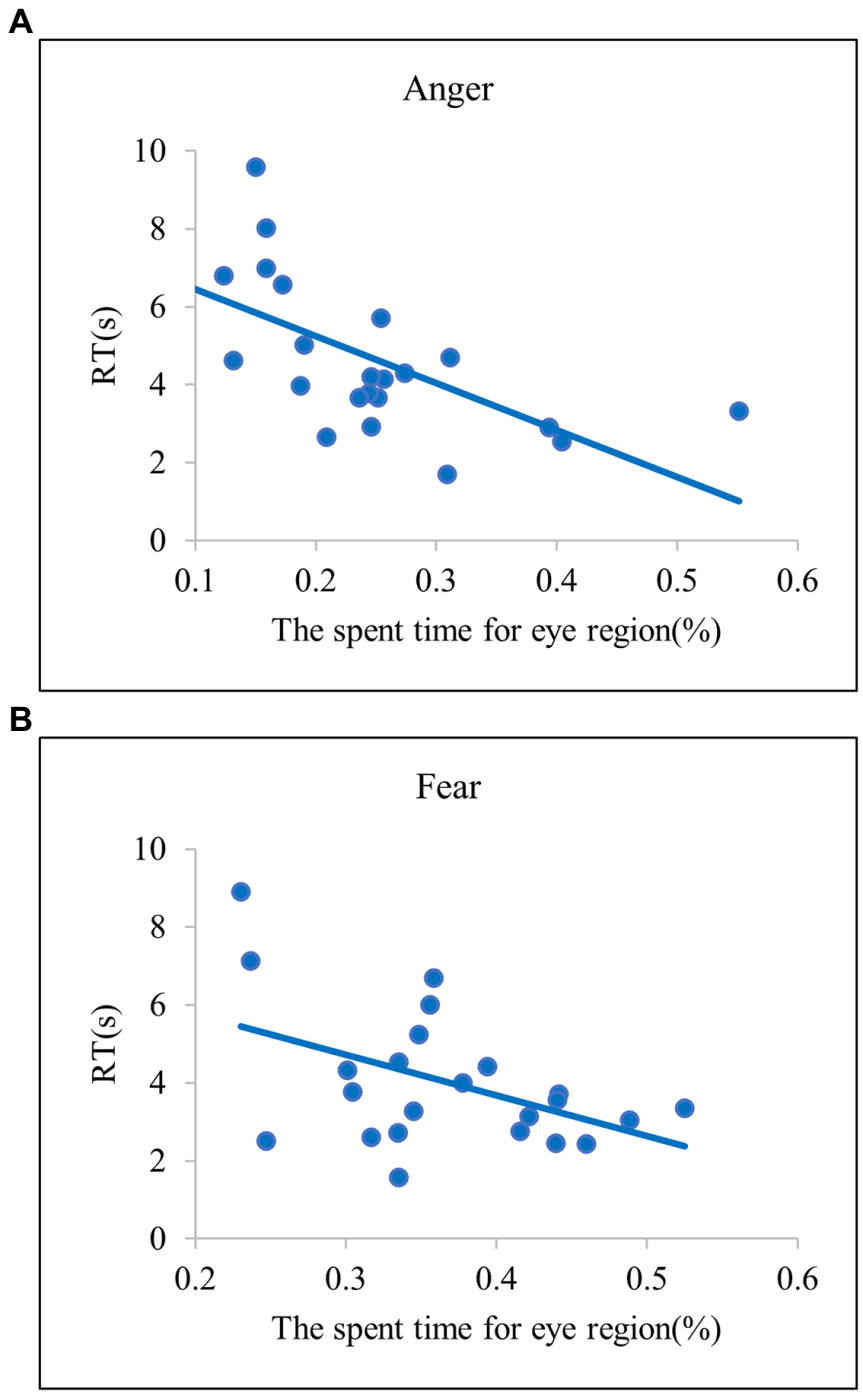
Scatter plots of the relationships between the spent time for eye region and RT in **(A)** Anger and **(B)** Fear. The scatter plots indicate that anger and fear have diagnostic regions in the eye areas.

**Figure 5:**
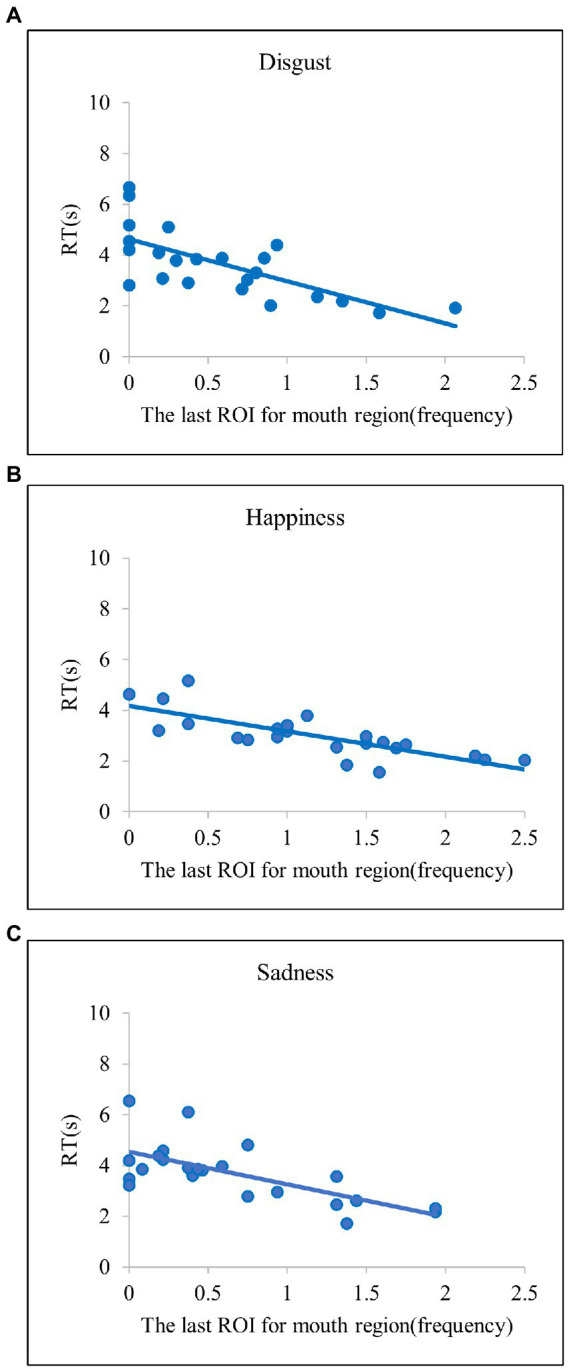
Scatter plots of the relationships between the last ROI for mouth region and RT in **(A)** Disgust, **(B)** Happiness, and **(C)** Sadness. The scatter plots indicate that disgust, happiness, and sadness have diagnostic regions in the mouth areas.

**Figure 6:**
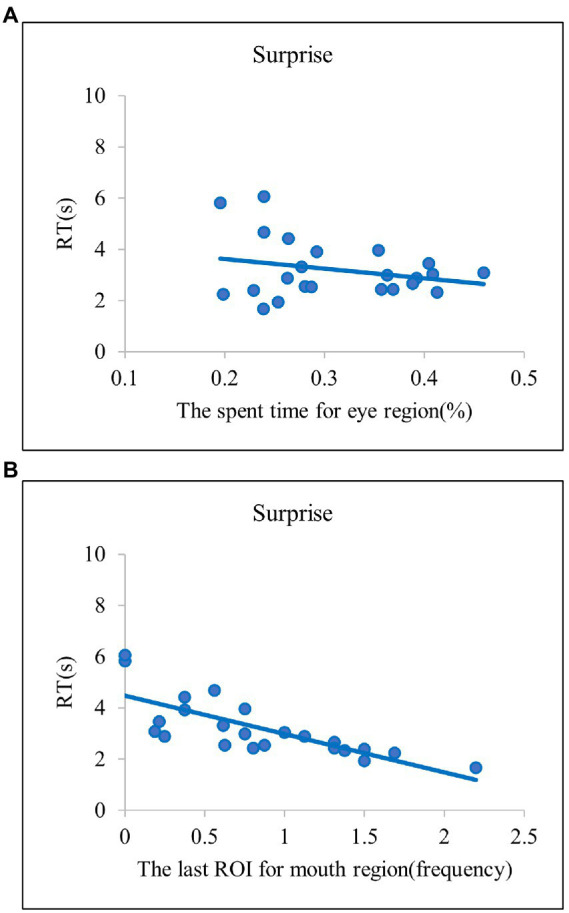
The scatter plots of the emotion of surprise. **(A)** the relationship between the spent time for eye region and RT in surprise. **(B)** the relationship between the last ROI for mouth region and RT in surprise. The scatter plots indicate that surprise have diagnostic regions in both the eye and mouth areas.

Overall, we identified reliable explanatory variables predicting efficient emotion recognition in both regression models in common. In the two models, the spent time for the eye region significantly explained efficient recognition for anger and fear emotions (Figure 4). These results indicate that, when the eye region is explored more frequently, the anger and fear emotions are correctly identified at a faster rate. For disgust and happy emotions, the last ROI for the mouth region significantly explained the efficient emotion recognition Figure 5A and B. Such results suggest that searching for the mouth region can significantly contribute to efficient emotion recognition performance for the disgust and happy emotions. Although there was no common variable revealing significant results for both models for sadness, the model based on the present study provided evidence such that searching for the mouth region could lead to faster recognition of sad emotion Figure 5C. Lastly, in the two regression models in common, both the spent time for the eye region and the last ROI for the mouth region significantly accounted for faster RTs for the surprise emotion Figure 6. We also conducted the same series of regression analyses for emotion recognition accuracy, but no significant results were found.

## Discussion

The present study aimed to identify the relationship between the exploration of emotion-specific diagnostic regions and efficient emotion recognition processing. Through a series of regression analyses, for the first time, we found that the exploration of emotion-specific diagnostic regions in each emotional expression can explain the RTs for accurate emotion recognition. Specifically, for anger and fear, exploration of the eye region significantly explained the fast responses to correct emotion recognition. On the other hand, for disgust and happiness, exploration of the mouth region explained the fast responses to correct emotion recognition. Likewise, for sadness, exploration of the mouth region explained the fast responses in emotion recognition, although the results were not consistent with prior findings on emotion-specific diagnostic regions. Finally, for the surprise emotion, the exploration of both the eye and mouth regions together can significantly explain fast emotion recognition. That is, the eye region was the most diagnostic for identifying angry and fearful facial expression. When viewers explored the eye region of the face more frequently, the response to emotion recognition was faster. Since the significant parameter for anger and fear (i.e., spent time) reflects how much and how long the specific regions are explored, the results imply that preferential exploration of the eye region contributes to the efficiency of accurate emotion recognition. On the other hand, for happy, disgusted, and sad emotions, the mouth region was the most diagnostic region for emotion recognition. The significant parameter for these emotions (i.e., the last ROI) reflects the regions that are visited when the correct emotion recognition is made. Thus, the results suggest that the mouth region contains the most emotion-specific information related to happiness, disgust, and sadness, leading to efficient emotion recognition processing. For the surprise emotion, both the eye and mouth regions were diagnostic regions for emotion recognition. When viewers explored the eye and mouth regions more frequently, surprise was recognized more quickly. In summary, the results of the current study demonstrate that the exploration of emotion-specific diagnostic regions contributes to more efficient processing of accurate facial emotion recognition.

The diagnostic regions identified in this study were consistent with previous studies that used various methods (Eisenbarth and Alpers, 2011; Birmingham et al., 2013, 2018; Schurgin et al., 2014; Wegrzyn et al., 2017; Neta and Dodd, 2018; Bodenschatz et al., 2019). While prior research adopted the MWT only for certain emotions (i.e., anger, fear, happiness, and disgust; Birmingham et al., 2013, 2018), the current study identified the diagnostic regions for all six basic emotions. Furthermore, contrary to the prior studies (Birmingham et al., 2013, 2018), here we conducted a series of regression analyses in order to discover predicting factors to explaining efficient emotion recognition processing for all six basic emotions. As a results, we found significant roles of the search patterns in the emotion-specific diagnostic regions on the efficient emotion recognition. Therefore, our study is significant in that it provides integrated understandings of the diagnostic regions for facial emotion recognition by applying the MWT in neurotypical young adults.

Furthermore, our results demonstrate the effects of diagnostic regions on the efficient processing of accurate emotion recognition for each of the six basic emotions. Thus far, there have been few evidence and inconsistent findings on the effects of diagnostic regions on emotion recognition with neurotypical young adults. However, we adopted the MWT, which could measure participants’ active explorations of faces and control the unattended visual information of the face and demonstrated how searching for diagnostic regions could affect efficient emotion recognition for the first time. Contrary to the methods that presented facial regions passively (e.g., hiding some parts of the faces), the MWT could capture the effects of rapid and active explorations of diagnostic regions on emotion recognition by providing an active searching environment for viewers. Furthermore, by controlling non-overtly attended visual information, the MWT could sensitively detect which visual information was explored for decoding specific emotions and the regions that were not attended.

For sadness, some of the prior studies argued that the eye region is the most diagnostic for sadness recognition (Birmingham et al., 2013, 2018). Inconsistent with these previous findings, our study found that the mouth was the most diagnostic region for recognizing sad emotions. Further, we found that explorations of the mouth region significantly explained the efficiency of correctly recognizing sad emotions. According to the Facial Action Coding System (FACS; Ekman and Friesen, 1978), which has been widely accepted in studies on facial emotions, sadness is expressed through the movement of muscles in the eyebrows and lips. In contrast, other emotions that can be detected with the eye area as a diagnostic region (e.g., fear, anger, and surprise) are expressed and recognized through the movement of the eye itself through the muscles of the upper and lower eyelids. In fact, the configuration of facial features for sadness is similar to that of emotions that have a diagnostic region in the mouth area (e.g., happiness, disgust). For example, the happy face includes little movement of the eye region, and the disgusted facial expression includes the movement of the eyebrows (as in the sad face). Thus, the mouth region is a more feasible facial area for differentiating sadness from the other emotions. The results of the present study offered new evidence for determining the diagnostic region for sadness, which contributed to emotion recognition by providing evidence that explorations of the mouth region explained the efficiency of recognizing sad faces.

It is noteworthy that different exploration variables explained each emotion recognition efficiency: the spent time and the last ROI. Specifically, for emotions with the eye region as a diagnostic region, the significant predictor was spent time on the eyes (i.e., anger, fear, and surprise). However, for emotions with the mouth region as a diagnostic region, the significant predictor for RTs was the last ROI on the mouth (i.e., disgust, happiness, sadness, and surprise). These findings could be attributed to the general exploration patterns of facial expressions. In fact, the results of ANOVAs and following *post-hoc* *t*-tests on the spent time showed that the spent time for the eye region was greater than the spent time for the mouth region for all emotions. Also, the results of ANOVAs on the last ROI revealed that the last ROI for the mouth region was greater than the last ROI for the eye region for all emotions except for anger. The spent time represented the amount of exploration toward the diagnostic regions and the last ROI represented the frequency of diagnostic regions where the participants visited when they made their emotion judgment decisions. Thus, although the accurate recognition of emotion was more efficient when the emotion-specific diagnostic regions were explored, there was a general tendency to explore more on the eye regions across all facial emotions in our study. Furthermore, while participants visited the eye regions more than the mouth area, they showed a tendency to terminate their exploration in the mouth region. Thus, significant differences in exploration for the eye region were revealed through the spent time, which reflected the overall amount of time spent focusing on the explored regions. However, significant exploration of the mouth region was well evidenced by the last ROI, which reflected how often the region was visited when viewers terminated the exploration. Such a general tendency to search for facial expressions of emotion has also been found in previous studies, which used an eye-tracking method (Schurgin et al., 2014; Bodenschatz et al., 2019). Considering that people make eye contact when they participate in social activities and that eye contact is related to the development of social cognition (Senju and Johnson, 2009), it is not surprising that there has been a general tendency to focusing more on the eye regions than to the mouth regions among neurotypical adults. Furthermore, it seemed that, in the recognition process for facial expressions, there was a general exploration pattern of searching for the eye regions first, followed by a search for the mouth region. Thus, future studies that include measures of the time course of exploration patterns in emotion recognition can elucidate whether such exploration patterns have an impact on the efficiency of facial emotion recognition.

Another point that is worthy to discuss is about the emotion recognition accuracies. In many previous studies, the accuracy was the highest for recognition of happy emotion (Hoffmann et al., 2010; Birmingham et al., 2013; Wegrzyn et al., 2017) compared to other emotions. However, in the current study, the highest mean accuracy was found in the surprise emotion although there was no significant statistical differences between the accuracies of happy and surprise emotions. In Birmingham et al. (2013), which applied the same methods as in the current study, the surprise emotion was not investigated. Thus, whether the accuracy of recognizing the happiness would be higher than that of the surprise in the previous study is undefined. Interestingly, in several previous studies with Korean participants, we found similar results on accuracies of happiness and surprise to the current findings (Kim et al., 2017c, 2022). In both previous studies, the accuracies of happy and surprise emotions were the highest and there was no reported statistical differences between the recognition accuracies of the two emotions. Furthermore, Kim et al. (2022) showed that the surprise emotion identified with the highest accuracy as in the current study. Thus, such results might represent the cultural differences on recognizing happy and surprise emotions, and future studies need to consider possible cultural differences in emotion recognition patterns.

The present may have some limitations with regard to measuring the exact amount of attention to facial regions. The blurring process in the MWT was adopted to prevent the confounding effects of extrafoveal information on emotion search. However, this could result in unnatural visual environments compared to the visual experiences in studies using an eye-tracking method. Furthermore, it is possible that participants might not focus on the window because their actual eye movements were not tracked. A method called the gaze-contingent paradigm (GC) was introduced to measure the actual visited regions on faces and more precisely control extrafoveal vision. The GC is similar to the MWT, except that the window is contingent on the viewer’s eye-gaze instead of the computer mouse (Caldara et al., 2010; Kennedy and Adolphs, 2010; Kim et al., 2017a,b). The GC is similar to the combination of the MWT and eye-tracking methods. However, additional equipment, such as an eye tracker, is required to track eye movement in GC. Considering the application of facial emotion recognition results to clinical populations as well as for developing children, the MWT may be a more practical and useful method than the GC. Furthermore, it is worth mentioning that a study comparing GC and MWT did not find significant differences between the two methods in terms of fixation proportion while viewing social scenes (Dalrymple et al., 2011).

Another possible limitation is restricted gender variability of participants. There are several studies reported sex differences (generally women’s superiority) on accuracy and RTs of facial emotion recognition (Saylik et al., 2018; Wingenbach et al., 2018). However, other studies showed evidence that there were no gender differences in the facial emotion recognition in young adults (Hoffmann et al., 2010; Abbruzzese et al., 2019). Still, there is a possibility that males and females may show different searching patterns for facial expressions. However, studies using similar methods to the current study reported very similar searching patterns to our results, although the previous studies included both male and female participants (Birmingham et al., 2013; Wegrzyn et al., 2017). As a matter of fact, we are currently conducting a follow-up study with children (both males and females), and the results in the study replicate our current findings on the emotion-specific diagnostic regions. Thus, we carefully expect insignificant gender differences on the emotion-specific diagnostic regions and their roles on emotion recognition performance, although the limited gender variability in the current study should be considered in the future study.

As mentioned previously, one of the advantages of the MWT is the convenience of implementing. Without additional apparatus such as an eye tracker, the MWT could be applied to anyone who is able to control the computer mouse and has normal vision. Therefore, the results of the present study can be easily applied to computerized training programs for individuals with emotion recognition deficits. For example, individuals with autism spectrum disorder (ASD) have deficits in facial emotion recognition, and their visual scan patterns are abnormal compared to those of typically developing individuals (Enticott et al., 2014; He et al., 2019; Liu et al., 2019). Furthermore, one of the well-known cognitive characteristics of people with ASD is weak central coherence: difficulty in synthesizing local information globally (Happé and Frith, 2006). Based on the weak central coherence theory, Ryan and Charragáin (2010) conducted a training program to teach children with ASD how to identify facial features expressing specific facial emotions and showed the significant effects of the intervention. The MWT can be applied to such training programs or fundamental research to examine the abnormal facial emotion recognition exploration patterns of people with neurodevelopmental disorders. We believe that the present study can be extended to different age groups and individuals with deficits in emotion recognition in future studies. Furthermore, a prior study applying the MWT to diverse age groups (Birmingham et al., 2013) showed that children’s exploration patterns differed from those of adults (especially for the eyes, mouth, and nose). Thus, although the current study set the ROI only for the eye and mouth regions based on prior diagnostic region studies with adult participants, future studies dealing with developing children will have to consider the ROIs of other facial features.

In summary, the current study provides evidence of emotion-specific diagnostic regions for all six basic emotions. Moreover, we believe that this current study provides a novel finding—chiefly, that searching for emotion-specific diagnostic regions can aid efficient emotion recognition processing in neurotypical adults. We believe that our methods and findings have ecological and practical value in developing assessments and interventions for facial emotion recognition processing in both clinical and developing populations with emotion recognition problems.

## Data availability statement

The datasets presented in this article are not readily available because of the sensitivity of the data. Requests to access the datasets should be directed to Dr. So-Yeon Kim, vicky47syk@duksung.ac.kr.

## Ethics statement

The studies involving human participants were reviewed and approved by the Institutional Review Board at Duksung Women’s University. The participants provided their written informed consent to participate in this study.

## Author contributions

MK: conceptualization, data curation, formal analysis, and writing-original draft preparation. YC: software and programming. S-YK: conceptualization, project administration, supervision, validation, draft-reviewing and editing, and funding acquisition. All authors contributed to the article and approved the submitted version.

## Funding

This work was supported by the National Research Foundation of Korea (NRF) grant funded by the Korea government (MSIT) (NRF-2020R1F1A1058200).

## Conflict of interest

The authors declare that the research was conducted in the absence of any commercial or financial relationships that could be construed as a potential conflict of interest.

## Publisher’s note

All claims expressed in this article are solely those of the authors and do not necessarily represent those of their affiliated organizations, or those of the publisher, the editors and the reviewers. Any product that may be evaluated in this article, or claim that may be made by its manufacturer, is not guaranteed or endorsed by the publisher.
